# Mixed Methods Pilot Study of Sharing Behaviors among Waterpipe Smokers of Rural Lao PDR: Implications for Infectious Disease Transmission

**DOI:** 10.3390/ijerph10062120

**Published:** 2013-05-24

**Authors:** Robyn Martin, Sahar D. Safaee, Khamphithoun Somsamouth, Boualoy Mounivong, Ryan Sinclair, Shweta Bansal, Pramil N. Singh

**Affiliations:** 1 Department of Epidemiology and Biostatistics, School of Public Health, Loma Linda University, Loma Linda, CA 92350, USA; E-Mails: robmartin@llu.edu (R.M.); sdsafaee@gmail.com (S.D.S.); 2 Department of Global Health, School of Public Health, Loma Linda University, Loma Linda, CA 92350, USA; 3 Center for Information and Education for Health, Ministry of Health, Vientiane, Lao PDR; E-Mails: ksomsamut@hotmail.com (K.S.); mounivong_99@hotmail.com (B.M.); 4 Department of Environmental Health, School of Public Health, Loma Linda University, Loma Linda, CA 92350, USA; E-Mail: rsinclair@llu.edu; 5 Department of Biology, Georgetown University, Washington, DC 20057, USA; E-Mail: shweta@sbansal.com; 6 Fogarty International Center, National Institutes of Health, Bethesda, MD 20892, USA; 7 Center for Health Research, School of Public Health, Loma Linda University, Loma Linda, CA 92350, USA

**Keywords:** infectious disease, waterpipe, mode of transmission, person-to-person, communicable

## Abstract

To date, the sharing behaviors associated with the homemade tobacco waterpipe used in rural areas of the Western Pacific Region have not been studied. Evidence from studies of manufactured waterpipes raises the possibility of infectious disease transmission due to waterpipe sharing. The objective of our pilot study in rural Lao People’s Democratic Republic (PDR) was to identify and measure the prevalence of waterpipe sharing behaviors. We first conducted ethnographic studies to investigate waterpipe-smoking behaviors. These findings were then used to develop an interviewer-administered household survey that was used in a sampling of waterpipe smokers from three villages of the Luang Namtha province of Lao PDR (n = 43). Sampled waterpipe smokers were predominantly male (90.7%), older (mean age 49, SD 13.79), married (95.4%), farmers (78.6%), and had completed no primary education. Pipes were primarily made from bamboo (92.9%). Almost all (97.6%) smokers were willing to share their pipe with others. At the last time they smoked, smokers shared a pipe with at least one other person (1.2 ± 0.5 persons). During the past week, they had shared a pipe with five other persons (5.2 ± 3.8 persons). The high prevalence of sharing behaviors among waterpipe smokers in rural Southeast Asia raises the possibility that this behavior provides important and unmeasured social network pathways for the transmission of infectious agents.

## 1. Introduction

The World Health Organization has reported that the Western Pacific Region has the highest global rate of cigarette smoking, with nearly two-thirds of men being current smokers [1]. Although the public health impact of cigarette smoking has been quantified in this region, the contribution of non-cigarette forms of tobacco use is only beginning to be studied and in some cases is virtually unknown. Throughout Southeast Asia, several ethnic groups (*i.e.*, Akha, Hmong, Khamu) living in communities as large as 90,000 have long smoked tobacco using homemade bamboo waterpipes [2]. In 2010, findings from the Global Adult Tobacco Survey of Vietnam indicated that there were an estimated 4.1 million tobacco waterpipe smokers in the nation [3]. Similar to cigarettes, recent findings also link pipe tobacco use to poverty in Vietnam [4]. In a 2006 national survey of Cambodia, the prevalence of tobacco pipe use among ethnic minorities living on the Lao-Cambodia border exceeded 30% in men and 48% in women [5]. Globally, there is a perceived “safety” associated with waterpipe smoking based on the belief that relative to cigarette smoking, the water filtration of the smoke in the pipe removes harmful toxicants [6]. Contrary to these popular beliefs, recent studies have shown that waterpipe smoking is associated with lung disease, cardiovascular disease [7], malignancy [8,9,10], adverse birth outcomes [8,11,12], and increased susceptibility to infectious agents [13,14].

**Figure 1:**
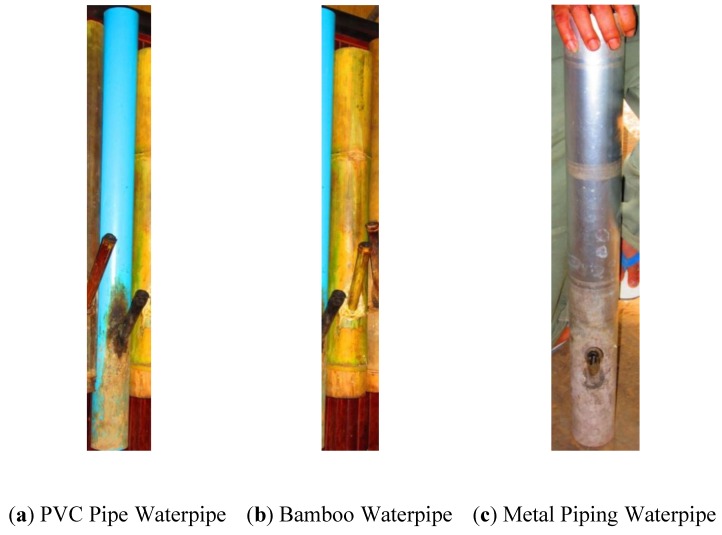
Homemade waterpipes used by study subjects

When considering the global health burden associated with tobacco waterpipe smoking, the common practice of multiple users sharing a waterpipe during a single session of smoking [15,16,17] is noteworthy in its potential creation of communicable disease health hazards. For the manufactured waterpipes long used in the Eastern Mediterranean Region, and now an emerging behavior in the West, the most common (90% of users) form of waterpipe smoking is a shared behavior that usually occurs through multiple hoses connected to the same source of filtered smoke [17]. In contrast, in rural areas of the Western Pacific Region, a homemade, cylindrical waterpipe (Figure 1) is used to smoke tobacco and the design does not include charcoal for burning the tobacco [2,18]. To date, the sharing behaviors of homemade waterpipe users in the Western Pacific have not been characterized in detail in the health literature.

Current data suggest that the use of a common mouthpiece or smoke convection path (*i.e.*, through a hose or bamboo pipe) by manufactured waterpipe users increases the risk of acquiring herpes simplex virus (HSV-1), Epstein Barr [19], *Helicobactor pylori* [14], hepatitis B, various respiratory infections, including bacterial meningitis [13], and periodontal conditions such as oral candida [15,20]. There have also been confirmed cases of tuberculosis spread through communal use of a waterpipe [21]. This adds to the evidence from South Asia and the surrounding regions linking tobacco smoking to an important burden of tuberculosis infections [13,21,22].

**Figure 2:**
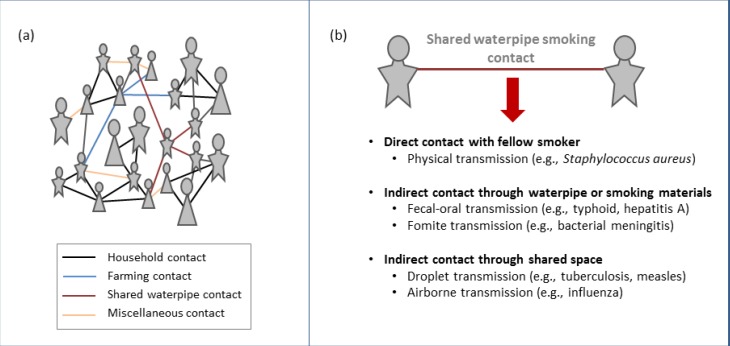
(**a**) Hypothetical network of potential disease-causing contacts in a rural Lao community, highlighting the possibility of additional links in an individual’s social network due to waterpipe smoking and sharing behaviors. (**b**) Possible pathways for the transmission of viral and bacterial pathogens due to waterpipe sharing behaviors.

In our present study, we focus on the homemade tobacco waterpipes of Lao PDR that tend to be made of bamboo, and less commonly of adapted pieces of plumbing pipe (PVC) or metal (Figure 1). The long tubular shape of the Laotian waterpipe provides ideal conditions for biofilm growth and the smoking mechanism of the waterpipe creates many potential pathogen-transmission pathways. When waterpipe smokers contaminate or come into contact with contaminated waterpipes or smoking materials, a potential exposure-pathway to fungal, bacterial, and viral pathogens is created between waterpipe smokers (See Figure 2). Due to poor access to modern sanitation, this population is particularly susceptible to transmission of infectious agents. In order to better understand the risk associated with the communal sharing practices of waterpipe smokers in the Western Pacific Region, it is imperative to first understand to what extent tobacco smokers are sharing waterpipes.

In this mixed methods pilot study of waterpipe smokers in rural Lao PDR our aims were as follows: (1) to identify behavior patterns associated with waterpipe use (*i.e.*, sharing, ownership); (2) to determine for each waterpipe user the number of persons who used the waterpipe they last smoked during the past 24 h and past 7 days; (3) to determine for each waterpipe user the concept of ownership of the pipe (by a household, by an individual) as well as willingness to share the waterpipe with others.

## 2. Methods

### 2.1. Study Population

During July 2011, as part of the validation study of a national tobacco survey [23], we worked with the Ministry of Health to select five villages from a rural district of the Luang Nam Tha province that were known to have a high prevalence of waterpipe smokers. In one of the villages, key informant interviews of five male waterpipe smokers (selected by systematic household sampling) were conducted by a Lao-speaking public health professional. This village was exclusively used to collect qualitative data. In the remaining four villages, we worked with multilingual interviewers to complete a systematic household sampling of waterpipe smokers and collect quantitative data via a paper-based survey. In each of the four villages, households had been enumerated as part of previous large-scale survey efforts and all enumerated households were eligible for selection. Study personnel (Ministry of Health interviewers, local tribal governance) were used to select all waterpipe smokers (all enumerated households were asked whether waterpipe smoking was occurring) from the first three villages and in the fourth village selection continued until a sampling target of more than 40 subjects was achieved. The selection criteria for all villages studied was that subjects were current waterpipe smokers aged 18 years and older who allowed investigators to examine the waterpipe they last smoked.

We had an overall response rate of 98% with a final sample of 43 subjects (four women, 39 men). Ministry of Health interviewers conducted the survey and there is a cultural tendency of the Lao to be compliant to government health surveys. Similar response rates have been found in large national surveys in Lao PDR (*i.e.*, Laos Reproductive Health Survey) [24]. Informed consent was obtained and ethics approval was obtained from the Ethics Committee of the Ministry of Health Vientiane Capital and the Institutional Review Board of Loma Linda University.

### 2.2. Qualitative Methods

A windshield survey of each village was conducted to obtain information about the environment around the villages. Windshield surveys are used as a qualitative research method in which researchers travel through a community to directly observe and assess factors that can contribute to community health. These observations can then be used to formulate interview or survey questions, make observations about environmental risk factors, and determine access to resources that promote community health (*i.e.*, fresh water, health clinics, *etc.*) [25]. From these surveys we observed type of waterpipe smoked, substances smoked in the pipes, types of houses in the village, potential water sources, and distance of the village from the main road. Key informant interviews were also used to gather qualitative data that provided a better understanding of waterpipe smoking behaviors in the community. Key informant questions were developed using information from secondary data-sources and through potential pathways that may increase risk of spreading disease. Questions focused on the type of pipe smoked, what substances the individual smoked, description of a typical smoking session, frequency of smoking, water source used for pipe, storage practices of smoked materials, eating habits, and personal hygiene. During each key informant interview, notes were taken for later analysis. After all interviews had been conducted, raw qualitative data was analyzed for themes using the open coding method. These themes were turned into codes, or labels, and placed into a codebook. These codes were then used to identify topics for further study in the survey. Several of the themes that were identified through the coding process were used to edit existing or create new questions for the survey.

### 2.3. Survey Design

The survey was designed based on: (1) the findings of a validation study where survey measures of tobacco use were compared to the findings from salivary cotinine and carbon monoxide testing [18]; (2) items adapted from the Global Adult Tobacco Survey (GATS) [3]; (3) a literature review of potential infectious disease transmission behaviors among users of smoked tobacco, and (4) a qualitative study described in the previous section.

The survey included items on demographics, tobacco (smoked, smokeless), behaviors associated with waterpipe use, environmental tobacco exposure and other environmental exposures, household and personal exposures, and current health status. The final survey was translated and back translated (between English and Lao) and checked for consistency by a Laotian public health professional. For data entry, a third party survey contractor fluent in Lao was used to complete double entry of the paper-based survey data into electronic format.

Three local interviewers (one Ministry level and two district level) conducted the surveys in each village. The Ministry level interviewer had extensive experience in demographic and health surveys and trained the provincial interviewers. Multi-lingual assistants from the subject’s villages were also used for subjects who could not understand the Lao language.

### 2.4. Statistical Analysis

Descriptive analyses were done on demographic, waterpipe type and usage behaviors, environmental exposure variables, and chronic symptoms. The confidence intervals for the number of shared users were determined using a non-parametric bootstrapping method (bias-corrected and accelerated) to account for small sample size and non-normal distributions. All analyses were performed using SAS version 9.3 (Cary, NC, USA) and SPSS version 20 (Armonk, NY, USA).

## 3. Results

### 3.1. Qualitative Study

Our findings from five key informant interviews of male waterpipe users from one village, and windshield surveys from five villages are described.

#### 3.1.1. Waterpipe Tobacco Characteristics and Storage

During five key informant interviews of male waterpipe users, two interviewees reported using loose tobacco in their waterpipes, but also reported their impression that the use of loose tobacco was declining due to the availability of manufactured cigarettes. The remaining three reported that they used commercial cigarettes in their tobacco waterpipes. Some of the interviewees loaded the waterpipe with crumbled cigarettes (excluding the filter). Others loaded the pipe by placing the entire cigarette, filter side down, in the bowl (stem on the side of the cylindrical pipe) of the waterpipe. When the entire cigarette was placed in the bowl, the smoker would hold the cigarette in the bowl of the waterpipe until they had completed their smoking session.

The two interviewees who used loose tobacco in their waterpipes stored their dried tobacco in a plastic bag or container due to humidity. They stated a belief that storage in such containers would prevent fungal growth in the tobacco that when smoked is “bad for your lung”. During windshield surveys of the five villages studied, we (R.M., S.D.S) also noticed that some men stored their loose tobacco in wood or metal boxes. Cigarettes were almost always stored in the packaging they were purchased in.

Across the five villages, three types of homemade waterpipes were seen in varying frequencies: bamboo, adapted PVC pipe, and adapted metal piping. Metal waterpipes tended to have been more labor intensive in their construction and were considered by the community to be more valuable.

#### 3.1.2. Sharing Behaviors

All five interviewees indicated that tobacco waterpipes in their region are frequently shared and waterpipe smoking is more often a communal activity than a solitary practice in their village. In fact, one of the five interviewees did not own a pipe, but did smoke a tobacco waterpipe daily by always borrowing a waterpipe. The interviewees indicated that, both personally and in the community, most waterpipe smokers had more than one pipe at home, and at least one in their hut in the field. Although our study did not specifically ask about the sharing of smoking materials (e.g., tobacco), we observed that ingredients were shared when an individual had their own pipe available to smoke but was missing ingredients. In this case, the individual borrowed smoking materials to smoke with their own pipe instead of using another pipe that was already loaded with ingredients. This also highlights the fact that waterpipes are shared for convenience, but an individual’s own pipe is preferred when available.

### 3.2. Quantitative Study

As shown in Table 1, the demographics of the sample indicate that the waterpipe smokers were predominantly male (90.7%), older (mean age 49, SD 13.79), married (95.4%), and farmers (78.57%) that had completed no primary education. In this sample, we found that almost all (97.1%) waterpipe users smoked daily. Findings from NATSC 2011 and national prevalence survey data being analyzed from Lao under the Fogarty/NIH also indicated that 1% or less of smokers in the region are less than daily smokers [26].

Although a wider variety of waterpipe types were observed during windshield surveys of the region, the quantitative survey findings shown in Table 2 reveal that the waterpipe smokers we studied primarily use homemade bamboo waterpipes (92.86%) as compared to metal (4.76%) and PVC pipe (2.38%). Images of the pipes actually used by some of the subjects are provided in Figure 1.

In this sample (n = 43), all waterpipe smokers claimed ownership of the pipe they last smoked, but most (97.6%) also indicated that they were willing to share their pipe with others (Table 3). In Table 3, we report that at the last time they smoked a waterpipe, each smoker had shared the pipe with at least one other person (1.21 ± 0.54 persons). During the past seven days, each smoker had shared a pipe with approximately 5 other people (5.24 ± 3.82).

**Table 1:** Demographics of 43 tobacco waterpipe smokers of rural Lao PDR (Luang Namtha Province, 2010).

	N (%)
**Education**	
No education	29 (69.1)
Primary school	12 (28.6)
Upper secondary	1 (2.38)
**Gender**	
Male	39 (90.70)
Female	4 (9.30)
**Marital Status**	
Never married	1 (2.33)
Married	41 (95.35)
Divorced/separated	1 (2.33)
**Ethnicity**	
Eui Mien	24 (55.81)
Lane Tane	3 (6.98)
Kouy	14 (32.56)
Lao Hauy	2 (4.65)
**Read/Write**	
Yes	12 (29.27)
No	29 (70.73)
**Vocational/Technical School**	
First level	3 (25.00)
Middle level	1 (8.33)
Other	1 (8.33)
Don’t Know	7 (58.33)
**Career**	
Government	1 (2.38)
Farmer	33 (78.57)
Homemaker	7 (16.67)
None	1 (2.38)
**Home Owner**	
Own	40 (95.24)
Live w/family & pay no rent	1 (2.38)
other	1 (2.38)
	**Mean ± (sd)**
**Age**	49 ± (13.79)

**Table 2:** Prevalence of Waterpipe Types.

Type of waterpipe	N (%)
Bamboo	39 (92.86)
Metal	2 (4.76)
PVC	1 (2.38)

**Table 3:** Waterpipe Sharing Practices.

	N (%)
**Owner**	
own & will share	41 (97.62)
own & will not share	1 (2.38)
	**Mean ± (sd)**
**Number of persons shared with during last smoking session**	1.21 ± (0.54)
**Number of persons shared with during last week**	5.24 ± (3.82)

## 4. Discussion

To date, our pilot study is the first to provide detailed qualitative and quantitative data on the pipe sharing practices of tobacco waterpipe smokers from rural regions of the Western Pacific Region. In a sample of 43 male and female waterpipe smokers of rural Lao PDR, our major findings are as follows: (1) most waterpipe smokers (97.6%) were willing to share their pipe with others, (2) at the last smoking session, smokers shared a pipe with at least one other person (1.21 ± 0.54 persons) (3) on a weekly basis, waterpipe smokers had shared a pipe with, on average, five other persons (5.24 ± 3.82 persons). The very high prevalence of sharing waterpipes in this sample raises the possibility that this behavior represents a significant route for transmission of the infectious disease pathogens that are highly prevalent in Lao PDR and the surrounding region (*i.e.*, tuberculosis, typhoid).

### 4.1. Evidence of Infectious Disease Transmission due to Sharing Among Waterpipe Users

The use and sharing of waterpipes have the potential to transmit bacterial and viral pathogens through airborne, droplet, fomite and fecal-oral routes of transmission. In Figure 2, we highlight some of these transmission pathways. The evidence, thus far, linking infectious disease transmission to waterpipe smoking is limited to case studies and small samples of those smoking manufactured waterpipes, but is convincing. Findings from samples that tested an association include: (1) a pulmonary tuberculosis cluster [21] found among young Australian adults (n = 149) where sharing a “bong” (a waterpipe with very similar construction to the Lao waterpipes) was associated with a two-fold increase in the likelihood of TB transmission (OR 2.22, 95% CI 0.96–5.17), and (2) a study of tuberculosis index patients and their household contacts (n = 112) in Storstroem County, Denmark found the same tuberculosis risk rates for household contacts of the index cases and shared waterpipe contacts of any infected case [27]. Additionally, there was the case report of a Russian student who was diagnosed with extremely drug resistant tuberculosis that was linked to a hookah lounge exposure before immigration to the United States [28].

When considering the possibility of a transmission pathway between the sharing of waterpipes and infectious disease, it should be noted that some of the findings may relate to fungal growth and other microbial contamination of the tobacco as the true point source rather than waterpipe itself. These microbial contaminants can become airborne or transfer from the tobacco to the surfaces of the waterpipe, fingers, or clothing, offering further routes of pathogen transmission. Such fungal growth in the tobacco used in these pipes includes *Aspergillus fumigatus*, *Penicillium marneffei*, *Fusarium proliferatum*, and several other microbiological contaminants [29,30,31]. A study performed in 2008 showed that tobacco smokers are also at increased risk of respiratory infection due to immunosuppression by several pathogens including *Streptococcus pneumonia*, *Neisseria meningitides*, *Haemophilus influenza* and *Legionella pneumophila* [13].

When comparing these findings to our pilot data from Lao PDR, we note that the design of the homemade waterpipe allows no exchange of pipe tips and involves greater contact between mouth, skin, and the point of inhalation than the more widely studied Eastern Mediterranean waterpipe (*i.e.*, “hookah”). Efforts to directly link the transmission of tuberculosis, herpes, typhoid and infectious pathogens through this behavior are needed in regional studies.

### 4.2. Implications for the Infectious Disease Burden in the Western Pacific Region

The Western Pacific Region is distinctive in having one of the highest global rates of both smoking and tuberculosis. A recent analysis by Basu *et al.* indicated that the Millennium Development Goals for TB control are delayed by at least two decades due to the high prevalence of smoked tobacco in the Western Pacific Region [32]. In Lao PDR, respiratory disease is still one of the leading causes of death in children under the age of five [33]. Recent studies linking the correlation between tobacco smoking and increased morbidity and mortality from respiratory infections show how dangerous waterpipe smoking can be in regions with high prevalence of infectious diseases [13,22]. Within these epidemics, our findings from Lao raise the possibility of transmission of infectious disease from smoker to smoker through social networks based on sharing (Figure 2). When waterpipes are shared, pathogens may spread from one host to another person through interactions with the pipe or smoking materials. The waterpipe sharing behavior also creates a social context in which airborne or droplet transmission due to proximity may occur with contacts not otherwise a part of an individual’s social network. For many years, infectious disease epidemiology has relied on social network modeling to track, control, and prevent the spread of infectious agents. Recent research on the mathematical modeling of these social networks has shown that an understanding of pathogen spread on local scales can lead to a better understanding of infection dynamics on larger spatial scales [34,35,36,37]. Tracking the sharing behaviors of waterpipe smokers may allow public health professionals to develop strategies to reduce infectious disease spread while decreasing the prevalence of tobacco smoking.

### 4.3. Limitations

Due to the small sample size (n = 43) of this pilot study of adults in the Luang Nam Tha Province, findings should be viewed as a reference for generating hypotheses to be investigated in large representative samples. We have focused on how sharing of a tobacco waterpipe may increase the potential for transmission of infections. Other factors that may increase risk of pathogen transmission include: tobacco handling and storage, cleaning of the waterpipe, hygiene practices, and environmental factors such as access to clean water, exposure to livestock, and availability of modern sanitation.

It is also relevant that we have only described the effects of tobacco in the waterpipes and did not find evidence of other additives during key informant interviews. During environmental sampling of the quantitative study sample (n = 43), however, we noticed that some subjects did add bamboo to the tobacco before smoking it. When asked about the reason for the added bamboo, one subject stated that “the tobacco was too strong and irritating to the lungs” when smoked alone, and the bamboo was added to make the smoke less irritating.

## 5. Conclusions

The findings from a mixed methods pilot study of waterpipe smokes in rural Lao PDR identify an extremely high prevalence of sharing behaviors that can potentially transmit infectious disease pathogens. Further efforts to identify the mode of transmission and measure the public health burden of this behavior are needed in this region where high rates of tobacco and infectious respiratory disease are occurring.
